# Acquisition of object-robbing and object/food-bartering behaviours: a culturally maintained token economy in free-ranging long-tailed macaques

**DOI:** 10.1098/rstb.2019.0677

**Published:** 2021-01-11

**Authors:** Jean-Baptiste Leca, Noëlle Gunst, Matthew Gardiner, I. Nengah Wandia

**Affiliations:** 1 Department of Psychology, University of Lethbridge, Lethbridge, Alberta, Canada T1K 3M4; 2 Faculty of Veterinary Medicine, Udayana University, Bukit Jimbaran, Bali, Indonesia

**Keywords:** token exchange, bartering, economic behaviour, symbolic tool, material culture, ecological validity

## Abstract

The token exchange paradigm shows that monkeys and great apes are able to use objects as symbolic tools to request specific food rewards. Such studies provide insights into the cognitive underpinnings of economic behaviour in non-human primates. However, the ecological validity of these laboratory-based experimental situations tends to be limited. Our field research aims to address the need for a more ecologically valid primate model of trading systems in humans. Around the Uluwatu Temple in Bali, Indonesia, a large free-ranging population of long-tailed macaques spontaneously and routinely engage in token-mediated bartering interactions with humans. These interactions occur in two phases: after stealing inedible and more or less valuable objects from humans, the macaques appear to use them as tokens, by returning them to humans in exchange for food. Our field observational and experimental data showed (i) age differences in robbing/bartering success, indicative of experiential learning, and (ii) clear behavioural associations between value-based token possession and quantity or quality of food rewards rejected and accepted by subadult and adult monkeys, suggestive of robbing/bartering payoff maximization and economic decision-making. This population-specific, prevalent, cross-generational, learned and socially influenced practice may be the first example of a culturally maintained token economy in free-ranging animals.

This article is part of the theme issue ‘Existence and prevalence of economic behaviours among non-human primates'.

## 1. Introduction

The token exchange paradigm is an appealing and heuristically powerful system used to investigate the existence of economic behaviour in non-human primates and to explore the evolutionary origins and developmental pathways of human monetary systems. Like a symbolic tool, a token is an inherently non-valuable (e.g. inedible or non-nutritive) object that acts as a secondary conditioned reinforcer; indeed, it only acquires an instrumental or functional value through the arbitrary associations made with the goods (e.g. food) or services (e.g. social or sexual favours) it is conventionally exchanged for in a barter-like situation, even though the object bears no iconic relation to its referent [1,2]. The tokens used by primate subjects in such experimental studies physically resemble human coins, and the act of trading tokens is similar to that of exchanging money for other commodities. The token exchange paradigm has shown that several species of monkeys and great apes can use tokens to request specific food rewards. This line of research provides insights into the cognitive underpinnings of economic behaviour in non-human primates [3].

However, most of these experimental procedures involve human-induced exchanges with relatively small samples of individually trained, laboratory-bred subjects. During the experiments, these subjects (i) are typically placed in isolation from their conspecifics and their other daily activities, (ii) exchanged in constrained environments characterized by a lack of alternative response options, and (iii) received small rewards for the correct actions ([1,2,4,5], but see [6–8]). These conditions markedly contrast with real-world human economic behaviours that offer many different formats and variants, often occur over extended periods of time, are spontaneously engaged in by a very heterogeneous population, use a range of symbolic currencies and are influenced by a rich social context [3,9].

In this respect, the external and ecological validities of the currently available token exchange paradigm could be put into question. This is not to say that the results obtained from the current studies suffer from a complete lack of validity, or that the results may not be informative about some contexts. However, the actual impact of conducting these experiments in these artificial conditions is unknown and needs to be investigated. One way to do that is to study more externally and ecologically valid systems of economic behaviours in non-human primates, and then to critically examine the generalizability of findings from laboratory models. This approach should provide a more solid platform for conducting comparative economics research and shed light on the evolution of human monetary systems [3].

Around the Uluwatu Temple in Bali, Indonesia, a large free-ranging population of long-tailed macaques (*Macaca fascicularis*) spontaneously and routinely engage in token-mediated bartering interactions with humans. These interactions occur in two phases: after stealing inedible and more or less valuable objects (e.g. pairs of glasses, hats, empty bags) from temple visitors, the macaques appear to use them as tokens, by returning them to humans in exchange for a certain number/type of food rewards proffered by the temple staff [10]. In many respects, these naturally occurring token-robbing and token/reward-bartering interactions are reminiscent of the token exchange paradigm experimentally implemented by researchers in captive settings, in which the symbolic value of a token lies in the quantity and quality of the food reward gained in return [2,8–15]. Our research project is the first to explore the cognitive and behavioural mechanisms underlying the *spontaneous* expression of token-mediated bartering interactions in *naturalistic* circumstances (i.e. the macaques initiate token-robbing interactions without any encouragement from humans). These interactions are at least partially monkey-driven (i.e. even though token/reward-bartering interactions depend on the willingness of human barterers to exchange, the macaques can choose to barter or not), and exhibited by a large number of free-ranging individuals [10].

Recent reports on robbing/bartering in the Uluwatu macaques have shown that this behaviour was (i) population-specific, because it was not observed in other anthropogenically influenced populations of Balinese macaques where it would be ecologically possible, (ii) frequent (7.8 robbing events/hour and 3.3 bartering events/hour), (iii) prevalent (49.3% of identified population members performed robbing behaviour and 35.6% performed both robbing and bartering behaviours), (iv) performed by individuals from all age/sex classes in the five neighbouring social groups of this population, (v) characterized by substantial intrapopulational intergroup variation in its frequency and prevalence (but not success), which was explained by anthropogenic influences (i.e. differential environmental opportunities to interact with temple visitors), and to a lesser extent, demographic correlates (i.e. the behaviour was more frequent and more prevalent in groups with higher ratios of (sub)adult males, but not group density), (vi) a behavioural candidate for the process of ‘cultural zones' (in which neighbouring social groups share space, information and behavioural practices) via intergroup observational learning or intergroup transfers of male group members, (vii) socially influenced and synchronized in its group-level expression, following a behavioural contagion-like effect, owing to response facilitation and possibly conformity-biased learning, (viii) not influenced by model-based biases (i.e. dominance rank, age, experience and success of the demonstrator), and (ix) an established practice in this population, probably passed on cross-generationally for at least 30 years, though detailed information on its origins is lacking [10,16]. However, we do not know whether this behaviour is learned through individual practice (i.e. via experiential trial-and-error learning) and underlain by economic decision-making processes—that is, subjected to robbing/bartering payoff maximization as would be suggested by clear behavioural associations between value-based token possession and quantity or quality of food rewards rejected and accepted by the monkeys.

This study aimed to fill this knowledge gap by testing three hypotheses pertaining to the acquisition and skillful performance of robbing and bartering interactions in the Uluwatu population of long-tailed macaques. First, the ‘experiential learning' hypothesis posits age differences in robbing/bartering success. Indeed, each of the two aforementioned phases (i.e. robbing and bartering) comprises several successive behavioural steps or strategies that must all be appropriately completed by the monkey for the phase to be considered successful. Both token-robbing and token/reward-bartering are challenging tasks for a monkey and require specific perceptual learning, sensorimotor coordination and cognitive skills. From juveniles to subadults to adults, we predicted (i) an increase in token-robbing success (i.e. the monkey sequentially stared at a temple visitor, approached and snatched a token from this person, and held the token, while stepping aside; *Prediction 1a*), (ii) an increase in token/reward-bartering success (i.e. the monkey waited for a human barterer and returned the undamaged token in exchange for at least one food reward; *Prediction 1b*), and (iii) more negotiated successful token/reward-bartering sequences (i.e. the monkey only returned the token after being proposed more food rewards, or after rejecting more food rewards, or after accepting a type of food reward different from the one(s) previously rejected; *Prediction 1c*).

Second, assuming a hierarchical scale of values attributed by humans to different tokens (with more valuable tokens leading to more bartering attempts by the temple staff), the ‘value-based token selection' hypothesis posits age differences in the selection of higher-valued tokens by the monkeys during the token-robbing phase. From juveniles to subadults to adults, we predicted an increase in the relative selection of higher-valued tokens available among *all* the temple visitors present on the site (*Prediction 2a*). When two tokens of different values were available on a *given* temple visitor, we predicted that subadult and adult monkeys would preferentially select the higher-valued token, whereas no significant difference was expected in juvenile monkeys (*Prediction 2b*). We predicted a similar age difference in the value-based token selection during field experiments, after controlling for token accessibility and lateral bias (*Prediction 2c*).

Third, assuming that economic decision-making requires cognitive maturity, the ‘robbing/bartering payoff maximization' hypothesis posits clear behavioural associations between value-based token possession and quantity or quality of food rewards rejected and accepted by subadult and adult monkeys (i.e. hypothetically the most skillful and selective individuals) during the token/reward-bartering phase. In other words, these individuals should respond differently to different token values, and use the token/reward-bartering phase to obtain either more food rewards or a more preferred food reward when they selected a higher-valued token. In terms of reward quantity, subadult and adult subjects should (i) wait to be proposed more food rewards (*Prediction 3a*), and (ii) accumulate more than one of the proposed food rewards (*Prediction 3b*) before returning the token when it is a higher-valued token. In terms of reward quality, they should (i) reject more low-preferred food rewards before returning the token when it is a higher-valued token (*Prediction 3c*) and (ii) more likely end a successful token/reward-bartering sequence by returning the token in exchange for a type of food reward different from the one(s) previously rejected when it is a higher-valued token (*Prediction 3d*). Conversely, subadult and adult subjects should more likely end a successful token/reward-bartering sequence by returning the token in exchange for a low-preferred food reward when it is a lower-valued token (*Prediction 3e*).

## 2. Methods

### (a) Study site and population

The Uluwatu Temple, located in a dry agricultural landscape of southern Bali, Indonesia, is a Hindu temple complex used by Balinese communities for daily religious ceremonies. It is also one of the most famous touristic spots on the island, visited by 1.5 million tourists in 2015 [16]. A population of long-tailed macaques has lived in this anthropogenic habitat for decades [17]. In April 2016, the study population comprised 333 individuals (55 adult males (older than 6 years), 21 subadult males (4–6 years), 79 adult females (older than 3.5 years), 20 subadult females (2.5–3.5 years), 94 juveniles (1–4 years for males and 1–2.5 for females) and 64 infants (younger than 1 year) split up into five neighbouring social groups with overlapping home ranges [16]*.* The Uluwatu macaques were provisioned daily with a variety of fruits and vegetables provided by the temple staff, and fully habituated to human presence. Overall, this habitat is a human-dominated area characterized by highly frequent interactions with (local and tourist) people and a very low predation pressure [16].

### (b) Data collection and scoring

Observational data were based on the spontaneous expression of token-robbing and token/reward-bartering interactions between monkeys and humans, with no intervention from the researchers. These data were collected by using the combined ‘behaviour-dependent sampling' rule and the ‘continuous recording' rule [18]. Behaviour-dependent sampling typically applies to data collection on ‘conspicuous' or ‘attention-attracting' behaviours [18, p.87]; these are characteristics that fit token-robbing and token/reward-bartering interactions, particularly because they usually occur within a few wide and open areas located around Uluwatu temple, where visitors are allowed and visibility for the researchers was good [10,16]. All observational data were continuously video-recorded from September 2015 to August 2016 (over 273 observation days) by two observers, using handheld camcorders (Sony HDR-PJ670). They typically walked across these open areas, regularly scanned the monkeys and their potential human targets around them, and started video-recording when a monkey stared at a prospective human target while approaching this person within 5 m. A prospective human target was defined as a temple visitor wearing or carrying around at least one inedible object that was more or less likely to be subsequently exchanged for food if stolen; examples of worn tokens included sun/eyeglasses and flip-flops, whereas examples of tokens carried around included cell phones and empty camera bags.

For each robbing/bartering event, one of the two observers video-recorded the complete token-robbing attempt and token/reward-bartering attempt, including the identity of the monkey being involved, the object (i.e. prospective token) being selected by the monkey, the number and type of other potential tokens directly accessible to the monkey (i.e. held or carried around by the human target), and the number and type of food reward(s) being (i) proposed/tossed to the monkey by an accustomed temple staff member typically located within 3 metres, (ii) ignored/rejected by the monkey with a ‘turning down' hand/arm gesture, and (iii) accepted/accumulated by the monkey by grabbing/cuddling the food reward(s). Because the monkeys were highly habituated to humans, most video-recordings were collected at close range (2–5 m) without disturbing the animals. Data collection started when the two observers reached high inter-observer reliability, as measured by the index of concordance (*C*) for monkey identities (*N* = 390 samples, *C* = 0.96) and age–sex class identification (*N* = 350, *C* = 0.93), and distances in metres (*N* = 200, *C* = 0.91) [16,19].

Even though these environmental conditions and behavioural sampling techniques allow for a majority of the token-robbing and token/reward-bartering interactions to be accurately and consistently recorded from start to finish, they did not provide *absolute* behavioural frequencies (i.e. number of events/behaviours per observation time unit) because the study site was too large for all robbing/bartering occurrences to be recorded at any time. However, this data collection method allowed us to quantify all the other variables used in this study (i.e. individual percentages of robbing/bartering successful attempts; number and type of object(s) being available on, and selected from, a human target; individual average numbers of food rewards being proposed, rejected, and accepted) because these measures were based on the *relative* behavioural frequencies calculated from a total of recorded events. All these variables were obtained from video-scoring. This task was conducted by N.G., who transcribed all the video-recorded robbing/bartering interactions onto an Excel spreadsheet, with each aforementioned variable being coded to the second. To measure intra-coder reliability, N.G. transcribed twice a total of 6.2 h of video-recordings, involving 158 token-robbing attempts and 66 token/reward-bartering attempts, which represents 3% of the total numbers of token-robbing attempts and token/reward-bartering attempts included in the analyses of token-robbing success and token/reward-bartering success, respectively. The comparison of the two transcriptions for all the other variables analyzed in this study yielded a high score of coder consistency (mean Cohen's κ coefficient: *k* = 0.89 ± 0.05 [19]).

### (c) ‘Experiential learning' hypothesis

We used individual percentages of successful token-robbing attempts and successful token/reward-bartering attempts as valid measures of experiential learning by conducting cross-sectional analyses based on age differences. To calculate these percentages, we used each monkey subject as its own control by distinguishing for each attempt two possible outcomes—successful or unsuccessful—out of the total number of attempts for each subject.

There was a four-stepped sequence of appropriate behaviours and environmental conditions leading to a successful token-robbing outcome: the monkey (i) stared at a prospective human target, (ii) inconspicuously approached the human target within 5 m, (iii) snatched the object from the human target (monkey–human body contact may or may not occur), and (iv) stepped aside while gripping the token. If any of these behavioural steps was not appropriately completed by the monkey, the token-robbing attempt was considered unsuccessful. To be included in the analyses of token-robbing outcome, an individual had to be involved in at least four token-robbing attempts (mean number of token-robbing attempts/individual ±s.d. = 53.5 ± 76.9). We believe that this minimum number of four attempts per individual made the distinction between unsuccessful and successful outcomes valid, while allowing us to reach a sample size for the ‘juvenile' age class that was conducive to statistical analysis.

Following a successful token-robbing attempt, there was a three-stepped sequence of appropriate behaviours and environmental conditions leading to a successful token/reward-bartering outcome: the monkey (i) held onto the stolen object and remained present in the area for a potential token/reward-bartering attempt involving a human barterer (i.e. typically one of the accustomed temple staff members, or occasionally the human target), instead of fleeing with an object that only acquires a value for the monkey during the bartering process, (ii) engaged in a token/reward-bartering interaction, which may involve accumulating several food rewards before returning the token, or even discarding a (a number of) less preferred food reward(s) in anticipation of receiving a (number of) more preferred food reward(s), and (iii) refrained itself from damaging the token and returned it in good condition, because if the token was damaged by the monkey, the bartering interaction may not occur or may be interrupted. If any of these behavioural steps was not appropriately completed by the monkey, the token/reward-bartering attempt was considered unsuccessful. To be included in the analyses of token/reward-bartering success, an individual had to be involved in at least four token/reward-bartering attempts (mean number of token/reward-bartering attempts/individual ± s.d. = 31.6 ± 39.0).

### (d) ‘Value-based token selection' hypothesis

Our observational data showed six types of tokens selected (i.e. either missed, if the token-robbing attempt was unsuccessful, or taken, if it was successful) by the Uluwatu monkeys: (i) empty containers (e.g. phone cases, camera bags, plastic bottles; *N*_missed_ = 260, *N*_taken_ = 287); (ii) accessories (e.g. hairpins, keyrings, bag-charms; *N*_missed_ = 171, *N*_taken_ = 270); (iii) hats (e.g. headgear, caps, crowns, veils, scarves; *N*_missed_ = 181, *N*_taken_ = 257); (iv) shoes (e.g. flip-flops, heeled sandals; *N*_missed_ = 897, *N*_taken_ = 902); (v) pairs of glasses (e.g. eyeglasses, sunglasses; *N*_missed_ = 351, *N*_taken_ = 1773); and (vi) electronic devices/wallets (e.g. cell phones, cameras, tablets, purses; *N*_missed_ = 48, *N*_taken_ = 77). Edible objects selected by the monkeys were not considered as tokens in our analyses.

To establish a hierarchical scale of values assigned by humans to these six types of tokens, we ranked them by increasing percentages of successful token-robbing attempts leading to token/reward-bartering attempts by humans (i.e. assuming more human-valued tokens should lead to more token/reward-bartering attempts by humans to retrieve the tokens from the monkeys). Our observational data showed three groups (each containing two types) of value-based tokens on the basis of how often humans wanted to engage in token/reward-bartering interactions with the monkeys: (i) low-valued tokens, seldom bartered by humans: empty containers and accessories (leading to bartering attempts in 8.4% and 16.3% of events, respectively); (ii) medium-valued tokens, often bartered by humans: hats and shoes (leading to bartering attempts in 74.3% and 75.8% of events, respectively); and (iii) high-valued tokens, almost always bartered by humans: pairs of glasses and electronic devices/wallets (leading to bartering attempts in 90.6% and 98.7% of events, respectively).

To assess the relative local availability in the six different types of tokens, we examined a sample of 84 video-recorded token-robbing events, randomly selected from our observational data, that featured 500 potential human targets. For each of these potential human targets, we scored the number and the type of potential tokens directly accessible to the monkeys. From this representative sample, the total number of available tokens we obtained was *N* = 1084, distributed across the six types of tokens as follows: (i) 127 empty containers; (ii) 107 accessories; (iii) 132 hats; (iv) 466 (pairs of) shoes; (v) 120 pairs of glasses; and (vi) 132 electronic devices/wallets. To assess the relative ease with which the six different types of tokens were stolen by the monkeys from human targets, we calculated, for each type of token, the average percentage of successful token-robbing attempts among all the subadult and adult monkeys (i.e. the most skilled robbers) that had each been involved in at least four token-robbing attempts targeting a given type of token in our observational dataset.

Experimental data were obtained from value-based token selection tests administered to a subset of 15 monkeys belonging to the three age classes (i.e. five juvenile, five subadult and five adult individuals) by three experimenters between September and December 2019. When each of these subjects was not surrounded by other monkeys, it was presented with a binary choice between a medium-valued token and a high-valued token, each proffered at opposing sides of a black plastic tray (30 cm × 20 cm × 4 cm). The tray was placed on the ground at 1.2 m from the tested monkey. The preferentially selected token was deemed to be the one the subject (first) grasped/took possession of. Each subject was tested across 16 trials comprising four randomly rotated sessions of four randomly rotated binary choices between a medium-valued token and a high-valued token (i.e. four trials with hat versus pairs of glasses, four trials with hat versus phone, four trials with shoe versus pairs of glasses and four trials with shoe versus phone). To control for lateral biases in grasping behaviour, token orientation (i.e. left/right) was balanced across trials. To control for experimenter biases, the experimenters fixed their gaze at a fixed point away from the proffered tokens during each trial. Most of these trials were followed by token/reward-bartering attempts by experimenters to retrieve the tokens. In terms of timing, and as much as possible considering field study constraints, the trials were homogeneously distributed within and across subjects, with no more than five trials being performed on a given subject within a day. Experimental data collection started when the three experimenters reached 100% of concordance for monkey identities.

### (e) ‘Robbing/bartering payoff maximization' hypothesis

For analyses on food rewards rejected and accepted by the monkeys during the token/reward-bartering phase, we only considered the food items that the monkey could reach/grab when proposed/tossed to them by the human barterers. To assess individual preferences across the three types of proposed food rewards (i.e. a raw egg, a small plastic bag containing a few pieces of fruit and a cracker), we used our observational data on spontaneously expressed token/reward-bartering interactions.

For a given monkey, we calculated three ‘food reward likeability' scores, one for each of the three types of proposed food rewards. When the three reward types were proposed during a token/reward-bartering sequence, we used two values: *X* was defined as the total number of times a certain reward type was the last one accepted by the monkey before returning the token and *Y* was defined as the total number of times this reward type was rejected by the monkey during token/reward-bartering sequences. The individual ‘likeability' score for a reward type was obtained by dividing *X* by (*X* + *Y*). The least-preferred type of food rewards for a given monkey was considered to be the one with the lowest ‘likeability' score.

### (f) Statistics

Because all our raw and (log, square-root and arsine-square-root) transformed data violated parametric assumptions, we conducted nonparametric tests. To test age differences in the percentage of successful token-robbing attempts, the percentage of successful token/reward-bartering attempts, and the average number of food rewards being proposed and rejected by the monkey, we used Kruskal–Wallis *H*-tests followed, when significant, by *post hoc* Mann–Whitney *U*-tests. To test whether the monkeys selected the six different types of tokens based on their relative local availability, we used a Spearman rank-order correlation test between the numbers of the six types of tokens available from our sample of 500 potential human targets and the numbers of the six types of tokens selected by the monkeys within our entire set of observational data (i.e. the sum of *N*_missed_ and *N*_taken_ for each type of token). To test whether the monkeys preferentially selected tokens that were easier to steal from humans, we used a Spearman rank-order correlation test between the percentage of types of tokens selected and the average percentage of successful token-robbing attempts among subadult and adult monkeys. To test within-individual preferential selection of low-valued, medium-valued and high-valued tokens, we used Friedman tests followed, when significant, by *post hoc* Wilcoxon signed-rank tests. To test within-individual preferential selection between two tokens of different values available on a given human target, we used Wilcoxon signed-rank tests. Value-based token selection tests of binary choices between a medium-valued token and a high-valued token were analyzed by using binomial tests with a test proportion = 0.50. Because all our predictions were directional, we conducted one-tailed tests. Statistical analyses were performed using the IBM-SPSS Statistics-26 analytical program. Significance levels were set at *α* = 0.05.

## 3. Results

### (a) ‘Experiential learning' hypothesis

**Figure 1.**
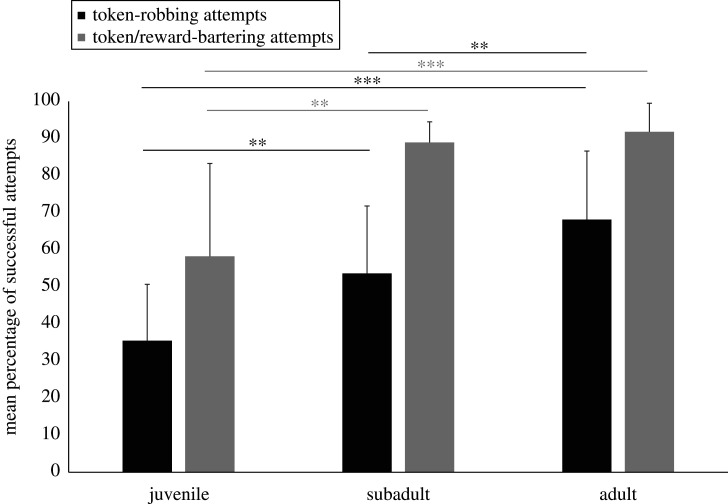
Mean (+s.d.) percentages of successful token-robbing attempts and token/reward-bartering attempts in juvenile, subadult and adult individuals (**p < 0.01, ***p < 0.001).

The percentages of successful token-robbing attempts were 39.1% (*N* = 376 attempts), 61.5% (*N* = 1829 attempts) and 68.8% (*N* = 3062 attempts) in juveniles, subadults, and adults, respectively. We found a statistically significant age difference in the percentage of successful token-robbing attempts (Kruskal–Wallis test, *H*_2_ = 35.8, *p* < 0.001). *Post hoc* pairwise comparisons revealed that subadults were more successful than juveniles (Mann–Whitney test, *N*_subadult_ = 20, *N*_juvenile_ = 17, *U* = 69.0, *p* = 0.002), adults were more successful than juveniles (*N*_adult_ = 60, *U* = 68.5, *p* < 0.001), and adults were more successful than subadults (*U* = 302.5, *p* = 0.001; figure 1). Therefore, we found a significant increase in token-robbing success from juveniles to subadults to adults; *Prediction 1a* was supported.

The percentages of successful token/reward-bartering attempts were 72.4% (*N* = 58 attempts), 89.4% (*N* = 689 attempts) and 92.0% (*N* = 1446 attempts) in juveniles, subadults and adults, respectively. We found a statistically significant age difference in the percentage of successful token/reward-bartering attempts (*H*_2_ = 22.3, *p* < 0.001). *Post hoc* pairwise comparisons revealed that subadults were more successful than juveniles (*N*_subadult_ = 13, *N*_juvenile_ = 10, *U* = 9.5, *p* = 0.001) and adults were more successful than juveniles (*N*_adult_ = 45, *U* = 20.0, *p* < 0.001). However, adults and subadults did not significantly differ in percentages of successful token/reward-bartering attempts (*U* = 213.0, *p* = 0.135; figure 1). Even though we did not find a significant increase in token-robbing success from juveniles to subadults to adults, *Prediction 1b* was partly supported owing to the difference between the former and the latter two age classes.

During successful token/reward-bartering sequences, we found statistically significant age differences in the average number of food rewards being proposed to the monkey (*H*_2_ = 15.1, *p* = 0.001), the average number of food rewards being rejected by the monkey (*H*_2_ = 13.9, *p* = 0.001) and the average number of food rewards being rejected by the monkey before accepting a different type of food reward to end the token/reward-bartering sequence (*H*_2_ = 8.7, *p* = 0.013). In each of these three variables, subadults scored significantly higher than juveniles (*N*_subadult_ = 14, *N*_juvenile_ = 9, *U* = 11.0, *p* < 0.001; *U* = 12.5, *p* = 0.001; *U* = 19.0, *p* = 0.004, respectively) and adults scored higher than juveniles (*N*_adult_ = 46, *U* = 41.5, *p* < 0.001; *U* = 49.0, *p* < 0.001; *U* = 87.5, *p* = 0.006, respectively). However, adults and subadults did not significantly differ in these scores (*U* = 301.0, *p* = 0.713; *U* = 313.5, *p* = 0.882; *U* = 305.0, *p* = 0.766, respectively). Even though we did not find a significant increase in the scores indicating negotiated successful token/reward-bartering sequences from juveniles to subadults to adults, *Prediction 1c* was partly supported owing to the difference between the former and the latter two age classes.

### (b) ‘Value-based token selection' hypothesis

In the observational data, the relative local availability in the six types of tokens and the numbers of the six types of tokens selected by the monkeys within our entire set of observational data were not statistically correlated (Spearman's correlation coefficient, *N* = 6, *R_s_* = −0.17, *p* = 0.742). Moreover, the average percentage of successful token-robbing attempts among all the subadult and adult monkeys (i.e. the most skilled robbers) and the percentage of types of tokens selected from human targets were not statistically correlated (Spearman's correlation coefficient, *N* = 6, *R_s_* = −0.26, *p* = 0.623).

In the observational data, the percentages of selection of low-valued, medium-valued and high-valued tokens were, respectively, 25.3, 47.9 and 26.8% in juveniles, 18.8, 38.9 and 42.3% in subadults and 17.2, 41.4 and 41.4% in adults. We did not find any statistically significant difference in the selection of the three differentially valued tokens by juveniles (Friedman test, *N* = 17, *χ*^2^ = 4.9, *p* = 0.085). However, we found statistically significant differences in the selection of the three differentially valued tokens by subadults (*N* = 20, *χ*^2^ = 8.5, *p* = 0.014) and adults (*N* = 60, *χ*^2^ = 21.4, *p* < 0.001). *Post hoc* pairwise comparisons revealed that subadults and adults preferentially selected high-valued over low-valued tokens (Wilcoxon tests, subadults: *z* = −2.0, *p* = 0.049; adults: *z* = −3.6, *p* < 0.001) and medium-valued over low-valued tokens (subadults: *z* = −2.0, *p* = 0.049; adults: *z* = −2.6, *p* = 0.010). There was no significant difference in the selection of high-valued or medium-valued tokens in subadults (*z* = −0.2, *p* = 0.872) and adults (*z* = −0.1, *p* = 0.997). Even though we did not find a significant increase in the relative selection of higher-valued tokens among all the prospective human targets from juveniles to subadults to adults, *Prediction 2a* was partly supported owing to the difference between the former and the latter two age classes.

However, when two tokens of different values were available on a given human target, we found that subadult and adult monkeys preferentially selected the higher-valued token (Wilcoxon tests, *N*_subadult_ = 12, *z* = −3.0, *p* = 0.003; *N*_adult_ = 12, *z* = −3.1, *p* = 0.002), whereas no significant difference was found in juvenile individuals (*N*_juvenile_ = 12, *z* = −0.775, *p* = 0.439). Therefore, *Prediction 2b* was supported.

In the experimental data, when two tokens of different values were available on plastic trays placed in front of a tested monkey, we found that subadult and adult subjects preferentially selected the higher-valued token (Binomial tests, subadults: *N*_medium-valued tokens selected_ = 12, *N*_high-valued tokens selected_ = 68, *p* < 0.001; adults: *N*_medium-valued tokens selected_ = 10, *N*_high-valued tokens selected_ = 70, *p* < 0.001), whereas no significant difference was found in juvenile subjects (*N*_medium-valued tokens selected_ = 35, *N*_high-valued tokens selected_ = 45, *p* = 0.314). Therefore, *Prediction 2c* was supported.

### (c) ‘Robbing/bartering payoff maximization' hypothesis

In this section about economic decision-making (i.e. testing behavioural associations between value-based token possession and quantity or quality of food rewards rejected and accepted), we focused on the most skilful and selective individuals during the token/reward-bartering phase, namely, subadult and adult monkeys. In terms of reward quantity, subadult and adult subjects waited to be proposed more food rewards before returning the token when it was a higher-valued token (i.e. a high-valued rather than a medium-valued token; Wilcoxon tests, *N*_subadult_ = 12, *z* = −3.1, *p* = 0.002; *N*_adult_ = 40, *z* = −3.0, *p* = 0.003). Low-valued tokens could not be used in these analyses because, by definition, they were typically not bartered by the temple staff. Still, *Prediction 3a* was supported.

We also found that the percentage of successful token/reward-bartering sequences when more than one of the proposed food rewards were accumulated by the monkey before returning the token was significantly higher when the monkey was holding a high-valued token than when it was holding a medium-valued token, both in subadult (Wilcoxon test, *N* = 10, *z* = −2.8, *p* = 0.005) and adult subjects (*N* = 29, *z* = −2.4, *p* = 0.015). Therefore, *Prediction 3b* was supported.

In terms of reward quality, and as expected, the average percentage of rejections, by adult subjects, of the least-preferred type of food rewards (when at least one reward among the least-preferred type was proposed during a given token/reward-bartering sequence) was significantly higher when they were holding a high-valued token, compared to a medium-valued token (Wilcoxon test, *N* = 22, *z* = −2.3, *p* = 0.024). However, this difference was not statistically significant in subadult subjects (Wilcoxon test, *N* = 10, *z* = −1.4, *p* = 0.153). Therefore, *Prediction 3c* was supported for adult, but not for subadult subjects.

Contrary to our expectations, there was no significant difference in the average number of food rewards being rejected by the monkey before accepting a different type of food reward to end the token/reward-bartering sequence when it was holding a high-valued token, compared to a medium-valued token, either in subadults (Wilcoxon test, *N* = 9, *z* = −1.2, *p* = 0.223) or in adults (Wilcoxon test, *N* = 29, *z* = −1.1, *p* = 0.280). Therefore, *Prediction 3d* was not supported.

Finally, subadult and adult subjects were significantly more likely to end a successful token/reward-bartering sequence by returning the token in exchange for the least-preferred type of food rewards (provided at least one reward among the least-preferred type was proposed during a given token/reward-bartering sequence) when they were holding a medium-valued token compared to when they were holding a high-valued token (Wilcoxon tests, *N*_subadult_ = 10, *z* = −2.2, *p* = 0.028; *N*_adult_ = 22, *z* = −2.1, *p* = 0.033). Therefore, *Prediction 3e* was supported.

## 4. Discussion

This field observational and experimental study of token-robbing and token/reward-bartering interactions in the free-ranging population of Balinese long-tailed macaques produced three main findings: (i) these behaviours need to be learned throughout juvenescence (i.e. until up to 4 years in this species) to be successfully performed; (ii) older monkeys preferentially selected tokens that were more valued by humans; and (iii) these more skilful and selective individuals appeared to make economic decisions, as evidenced by clear behavioural associations between value-based token possession and quantity or quality of food rewards rejected and accepted.

### (a) Experiential learning

As predicted, we found a significant increase in token-robbing success from juveniles to subadults to adults, whereas the main behaviour patterns required for the successful performance of token/reward-bartering interactions were already in place from around 4 years (i.e. in subadults). Likewise, the ability to engage in more negotiated successful token/reward-bartering sequences—during which the monkey only returned the token after being proposed more food rewards, or after rejecting more food rewards, or after accepting a type of food reward different from the one(s) previously rejected—was not fully acquired before the subadult stage.

These results lend some support to the ‘experiential learning' hypothesis, whereby token-robbing and token/reward-bartering interactions are multi-stepped and complex behavioural sequences requiring perceptual learning, sensorimotor coordination and cognitive skills (e.g. memory, associative learning) to be successfully performed; they are thus gradually acquired through extended individual practice during the juvenile period, in part via experiential trial-and-error learning. It is noteworthy to mention that the development of (sub)adult-level proficiency at robbing/bartering is not only dependent on skill learning (e.g. detection, sneaky approach, self-control), but may also be constrained by physical maturation. This is particularly true during the token-robbing phase that often involves monkey–human body contact and/or requires muscular strength when a monkey has to yank on a flip-flop still worn by an adult human. In these cases, the limited physical capabilities of juveniles, and the maturing bodies of subadults, may partly explain the significant increase in token-robbing success from juveniles to subadults to adults.

Primates are characterized by the longest juvenile period in relation to life span of all mammals [20]. According to the ‘needing-to-learn' hypothesis [20], prolonged juvenility is associated with behavioural patterns that necessitate acquiring a proportionally large amount of information and/or skills to reach adult competence before individuals become reproductively mature. These behaviours include extractive foraging techniques [21] and (socio-)sexual behaviour patterns [22]. Our study indicates that both phases of the robbing/bartering practice also required experiential learning to be fully mastered.

### (b) Value-based token selection

The first step in economic decision-making requires the cognitive ability to distinguish among different expected material values of a given symbolic currency (e.g. tokens, cash, virtual money). After showing that token selection was not significantly affected by token availability and the relative ease with which different types of tokens were stolen by the monkeys from human targets, our observational data revealed a marked age difference in how the monkeys responded to a human-based three-level hierarchy of valuable objects. When considering the token selection among all the prospective human targets (i.e. temple visitors with potential tokens available in a given area), juveniles did not show any preferential selection among low-valued, medium-valued and high-valued tokens, whereas subadults and adults preferentially selected high- and medium-valued tokens over low-valued ones. When two tokens of different values were available on a given human target, subadults and adults preferentially selected the higher-value token, whereas juvenile individuals did not show any significant difference. We found a similar age difference in the value-based token selection, after experimentally controlling for token accessibility and lateral bias.

These results support the ‘value-based token selection' hypothesis, positing age differences in the selection of higher-valued tokens by the monkeys during the token-robbing phase that are indicative of a developmental trajectory toward more strategic choices in more mature individuals. Subadult and adult monkeys (but not juveniles yet) have learned to map their token-robbing behaviours onto the hierarchical (and arbitrary) scale of values attributed by humans to different tokens: they preferentially selected tokens that were more likely to be exchanged for food (e.g. electronic devices, pairs of glasses) over other objects that were less valuable for humans and typically not worth bartering (e.g. empty camera bags, hairpins). Our findings are consistent with data obtained in other non-human primate species, showing that subadult and adult capuchin monkeys and chimpanzees correctly preferred a high-valued token over a low-valued token in an experimental bartering situation [1,11,23].

### (c) Robbing/bartering payoff maximization

The second step in economic decision-making requires the cognitive ability (i.e. mental processes involving associative learning and memorization) to respond differently to differentially valued tokens by trying to maximize one's payoff. We found evidence for such behavioural associations between value-based token possession and quantity or quality of food rewards rejected and accepted by subadult and adult monkeys (i.e. the most skilful and selective individuals) during the token/reward-bartering phase. They consistently and actively obtained either more food rewards or a more preferred food reward in exchange for a higher-valued token. They were also more likely to end a successful bartering interaction by accepting a less preferred food reward in exchange for a lower-valued token.

Our findings support the ‘robbing/bartering payoff maximization' hypothesis in that subadult and adult monkeys strategically responded to differentially valued tokens in their possession by adjusting the amount or type of food rewards they gained from the barters. The result showing that subadults (unlike adults) failed to significantly reject more low-preferred food rewards before returning a higher-valued token may be explained in terms of poorer temporal cognition or higher impulsivity (compared to adults): subadult long-tailed macaques may either not have yet acquired the cognitive capacity to anticipate the subsequent proffering of more preferred food rewards in this specific situation, or not be patient/self-controlled enough to wait for possibly more preferred food rewards.

Overall, our field observational data are in line with laboratory-based studies showing that several non-human primate species can (i) understand the effectiveness of tokens as secondary reinforcements to make simple calculations about quantities of reward, (ii) determine an item's value on the basis of its perceived utility (e.g. exchanging only a low-preferred reward for a tool necessary to reach a more preferred reward) and (iii) recognize the appropriate conditions in which a successful exchange could occur (e.g. presence/absence of the experimenter, safe/risky experimenter) [1,2,23–27]. Other cognitive skills and temperamental traits exhibited to varying extents by non-human primates engaging in token-aided economic behaviours include preference transitivity, self-control, delay of gratification, action planning and calculated reciprocity, because they may facilitate or constrain an individual's ability to make optimal economic decisions [1,5,23,28]. Even though these characteristics were not explicitly examined in this study, some of them will be the subject of our future observational and experimental investigations.

## 5. Conclusion

Token-robbing and token/reward-bartering are cognitively challenging tasks for the Uluwatu macaques that revealed unprecedented economic decision-making processes (i.e. valued-based token selection and payoff maximization) in a large monkey population living in an anthropogenically impacted habitat. This spontaneous, population-specific, prevalent, cross-generational, learned and socially influenced practice may be the first example of a culturally maintained token economy in free-ranging animals. The present naturalistic research setting represents a unique opportunity to study field economics and explore macroeconomic phenomena in non-human primates in environmental conditions that are more externally and ecologically valid than those provided by the traditional token exchange paradigm. Further experimental research on the Uluwatu macaques should make future cross-species comparisons of economic decision-making and symbolic tool use more relevant from an evolutionary perspective and may ultimately lead to a better understanding of the origins of autonomous monetary systems in humans [29].

## Data accessibility

The dataset ‘Acquisition of object-robbing and object/food-bartering behaviours: a culturally maintained token economy in free-ranging long-tailed macaques’ is available from the Dryad Digital Repository: https://doi.org/10.5061/dryad.wpzgmsbjh [30].
